# Developing Machine Learning Models for Cognitive Disorder Classification Using Administrative Health Data and Specialist‐Confirmed Diagnoses

**DOI:** 10.1002/alz70860_101252

**Published:** 2025-12-23

**Authors:** Gavin Thomas, Nils D. Forkert, Eric E. Smith, Geoffrey Messier, Mohammad Z I Chowdhury, Dallas Seitz

**Affiliations:** ^1^ Hotchkiss Brain Institute, University of Calgary, Calgary, AB, Canada

## Abstract

**Background:**

Mild cognitive impairment (MCI) and dementia affect a growing proportion of the aging population, placing significant demands on healthcare systems. While administrative health records (AHRs) capture broad healthcare data, their usefulness in identifying MCI and dementia is limited by a lack of expert‐confirmed diagnostic information. The Prospective Registry for Persons with Memory Symptoms (PROMPT) provides specialist‐confirmed diagnoses and detailed clinical information, creating a unique opportunity to validate machine learning (ML) models in real‐world settings. By linking PROMPT's registry data with population‐level AHRs, this study addresses current gaps in early detection and staging of cognitive decline, with implications for both clinical practice and health policy.

**Method:**

We analyzed data from 1,901 adults referred to a tertiary memory clinic in Calgary, Canada (2010–2023), with records linked to provincial Administrative Health Records (AHRs). Cognitive status was classified as “no objective impairment” (NOI), mild cognitive impairment (MCI), or dementia through comprehensive multidisciplinary assessments, including neuropsychological testing and specialist evaluations. Predictor variables derived from AHRs encompassed diagnostic codes, medication dispensations, and healthcare utilization patterns. We developed and compared multiple machine learning (ML) algorithms, including logistic regression, random forests, and ensemble methods, against previously established rule‐based approaches from a Canadian study. Model performance was evaluated using the F1 score to balance sensitivity and specificity, along with overall accuracy metrics.

**Results:**

Of the 1,901 participants (mean age 73 years, 52% male), 34% (*n* = 646) were diagnosed with dementia, 44% (*n* = 836) with MCI, 12% (*n* = 228) with NOI, and 10% (*n* = 191) received an 'uncertain' diagnosis. Initial analyses demonstrated expected patterns in cognitive assessment scores across diagnostic groups, including the Mini‐Mental State Examination (MMSE) and Montreal Cognitive Assessment (MoCA). Complete model performance metrics, including sensitivity, specificity, and area under the receiver operating characteristic curve (AUC), are currently being finalized and will be presented.

**Conclusion:**

Forthcoming results will clarify how specialist‐confirmed data from PROMPT can enhance ML‐based classification of MCI and dementia in AHRs. These findings have the potential to improve surveillance of cognitive disorders, inform healthcare resource allocation, and guide earlier interventions. By integrating advanced analytics with validated clinical information, this project could shape future approaches to dementia care in Canada and beyond.

